# Expression of SARS-CoV-2 receptor *ACE2* and the protease *TMPRSS2* suggests susceptibility of the human embryo in the first trimester

**DOI:** 10.1098/rsob.200162

**Published:** 2020-08-05

**Authors:** Bailey A. T. Weatherbee, David M. Glover, Magdalena Zernicka-Goetz

**Affiliations:** 1 Department of Physiology, Development and Neuroscience, University of Cambridge, Cambridge CB2 3EG, UK; 2 Department of Genetics, University of Cambridge, Cambridge CB2 3EH, UK; 3 Division of Biology and Biological Engineering, Caltech, Pasadena, CA 91125, USA

**Keywords:** TMPRSS2, SARS-CoV-2, receptor ACE2

## Abstract

While initially recognized as causing respiratory disease, the SARS-CoV-2 virus also affects many other organs leading to other complications. It has emerged that advanced age and obesity are risk factors for complications but questions concerning the potential effects on fetal health and successful pregnancy for those infected with SARS-CoV-2 remain largely unanswered. Here, we examine human pre-gastrulation embryos to determine the expression patterns of the genes *ACE2*, encoding the SARS-CoV-2 receptor, and *TMPRSS2*, encoding a protease that cleaves both the viral spike protein and the ACE2 receptor to facilitate infection. We show expression and co-expression of these genes in the trophoblast of the blastocyst and syncytiotrophoblast and hypoblast of the implantation stages, which develop into tissues that interact with the maternal blood supply for nutrient exchange. Expression of *ACE2* and *TMPRSS2* in these tissues raises the possibility for vertical transmission and indicates that further work is required to understand potential risks to implantation, placental health and fetal health that require further study.

## 1. Introduction

Beginning in December 2019, the novel coronavirus SARS-CoV-2 spread rapidly throughout the world population. It rapidly became clear that the virus has widespread effects upon many tissues and that thromboembolic events are the major cause of death. However, the consequences of infection for the unborn child are largely unaddressed.

Conflicting clinical reports have emerged regarding SARS-CoV-2 infection in neonates whose mothers were infected. Some studies have found no evidence for vertical transmission, while others found a percentage of neonates to be born with SARS-CoV-2 infection [1–3]. Although, some of these positive neonate cases could arise through viral transmission shortly after birth, at least one appears due to vertical transmission in the third trimester [3]. All these clinical studies have involved pregnant individuals whose SARS-CoV-2 infection was detected during the third trimester. Much less is known concerning the risks of vertical transmissionat at earlier stages. With these considerations in mind, we address here when in human development is the earliest stage at which SARS-CoV-2 receptor, angiotensin-converting enzyme 2 (ACE2), is first expressed and therefore when the human embryo could possibly become infected.

**Figure 1.**
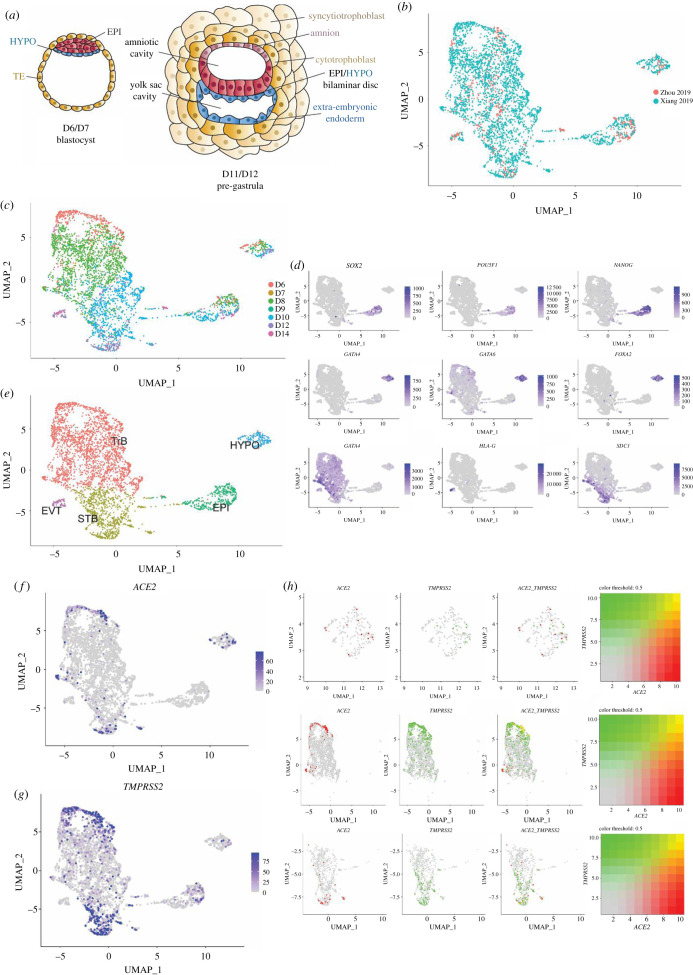
Expression of *ACE2* and *TMPRSS2* in the pre-gastrulation human embryo. (*a*) Schematics of the D6/7 late blastocyst and D11/12 pre-gastrula embryo. (*b*) uniform manifold approximation and projection (UMAP) of integrated published post-implantation RNA-sequencing datasets. (*c*) UMAP of integrated dataset labelled by timepoint. (*d*) UMAP of integrated dataset with expression levels of epiblast markers (*SOX2*, *POU5F1*, *NANOG*), hypoblast markers (*GATA4, GATA6, FOXA2*) and trophoblast markers (*GATA3* for pan-trophoblast, *HLA-G* for extravillous trophoblast, *SDC1* for syncytiotrophoblast) in TPM. (*e*) UMAP of integrated dataset labelled by cell-type cluster. (*f*) UMAP of integrated dataset with expression levels of *ACE2* in TPM. (*g*) UMAP of integrated dataset with expression levels of *TMPRSS2* in TPM. (*h*) UMAP of isolated integrated hypoblast, trophoblast and syncytiotrophoblast clusters with expression and co-expression of *ACE2* and *TMPRSS2*. EPI = epiblast, HYPO = hypoblast, TE = trophectoderm, TrB = Trophoblast, STB = syncytiotrophoblast, EVT = extravillous trophoblast.

Following fertilization, the single-cell zygote undergoes rapid cleavage divisions. On day 5 after fertilization, the blastocyst is formed, which will implant into the uterus on the following day (figure 1*a*). The early embryo at this stage is comprised trophoblast cells, which will give rise to the placenta to connect the developing fetus with the mother, hypoblast cells, which will give rise to the yolk sac, and epiblast cells which will give rise to both the amniotic epithelium (precursor of the amniotic sac) and the baby itself. Following implantation, the embryo undergoes a series of morphological and genetic changes. These include differentiation of large multinucleated syncytiotrophoblasts (derived from the trophoblast), which will contribute to the formation of connections with maternal blood vessels; differentiation of extravillous trophoblast, which will grow out from the placenta and penetrate into the decidualized uterus; and expansion of the hypoblast to form the primary yolk sac, important to establish the early embryonic blood supply (figure 1*b*). At day 14, the part of the epiblast undergoes gastrulation, during which the three germ lineages are formed that will give rise to all tissues in the baby. These stages of early development are complex and delicate. Despite the remarkable plasticity of the embryo, it is particularly vulnerable at this time with about 60% of pregnancies failing during these first 14 days of development [4,5].

Recently, a culture platform has been developed that permits the growth of surplus IVF human embryos up to day 14 [6–8]. This platform permits for the first time the exploration of gene expression patterns that provide signatures for all distinct cell types in the human embryo until the end of the second week of pregnancy. To gain a global view of gene expression in the different cell types of the human embryo, we have combined and analysed single-cell RNA-sequencing data available so far, including our own data [8,9], using the Seurat v3.1 integration protocol with 10 000 anchor points (figure 1*b,c*). We subjected this integrated data to clustering using principal component analysis and nearest neighbour approximation in 10 dimensions, which identified 5 clusters (figure 1*d*,*e*). The presence (+) or absence (−) of canonical marker gene expression identified these clusters as representing five cell types: a *SOX2+ POU5F1+ NANOG+* epiblast cluster; a *GATA4+ GATA6+ FOXA2+* hypoblast cluster; a *GATA3+ HLAG- SDC1-* trophoblast cluster; a *GATA3+ HLAG- SDC1+* syncytiotrophoblast cluster and a *GATA3+HLAG+SDC1-* extravillous trophoblast cluster (figure 1*d*,*e*).

SARS-CoV-2 entry into a cell involves the interaction of its spike protein with the cell's membrane-bound angiotensin-converting enzyme 2 (ACE2), which is cleaved by the transmembrane protease serine 2 (TMPRSS2) [10]. Blocking TMPRSS2 protease activity blocks ACE2-mediated entry of SARS-CoV-2 [10], suggesting that co-expression of both genes is required for infection. We, therefore, sought to determine the expression patterns of *ACE2* and *TMPRSS2* in the integrated RNA-sequencing dataset. Visualizing *ACE2* expression across all lineages revealed its enrichment in the day 6 and day 7 trophoblast (31.4% expressed), day 12 and day 14 syncytiotrophoblast (26.8% expressed) and hypoblast cells (21.5% expressed) (figure 1*f*). Visualizing *TMPRSS2* expression, revealed a similar, though more wide-spread pattern, in addition to expression in 32.8% of epiblast cells (figure 1*g*). We could detect the strong co-localization of expression of both genes 25.5% of the blastocyst trophoblast, as well as in 21.8% of syncytiotrophoblast (figure 1*h*). Co-localization in hypoblast cells was weaker, though still present in 7.2% of cells. Overall this analysis indicates that a subset of cells across different trophoblast and hypoblast lineages may be susceptible to SARS-CoV-2 infection.

The expression of *ACE2* and *TMPRSS2* in the hypoblast and syncytiotrophoblast may be hypothesized due to the role of ACE2 in attenuating ACE activity during the regulation of angiotensin cleavage in vasoconstriction [11]. Thus, ACE2 may be anticipated to function in the early development of the embryo's blood system enabling nutrient and oxygen exchange with the mother. After gastrulation, the yolk sac derived from the hypoblast gives rise to blood islands that will form capillaries close to maternal blood surrounding the embryo [12]. Later, the placenta, derived from the trophoblast, particularly syncytiotrophoblast, will be surrounded by maternal blood and will take over nutrient exchange. Thus, these derivatives of hypoblast and trophoblast come to exist close to and exchange nutrients with the maternal blood. The expression of *ACE2* and *TMPRSS2* in these tissues, therefore, raises the possibility of vertical transmission during these very early stages of human development when the majority of pregnancies remain unnoticed.

In addition to ACE2 and TMPRSS2, other potential SARS-CoV-2 receptors, proteases and cofactors for infection have been suggested, including BSG (CD147) [13] and NRP1 [14] as receptors, and CTSL as a key protease [15]. Importantly, each of these are also expressed in the pre-gastrulation embryo. *BSG* and *CTSL* are expressed in all tissues (supplementary figure 1a-b), while *NRP1* is enriched in the trophoblast (supplementary figure 1c). This suggests the possibility of multiple avenues through which SARS-CoV-2 may infect the early embryo.

The potential of viral infection through transmission of SARS-CoV-2 during early pregnancy via maternal blood can have implications for the success of implantation: future placental and fetal health. Our findings greatly extend another study indicating the expression of *ACE2* during later stages of placental development as well as in fetal organs such as the heart, liver and lung [16]. Importantly, these analyses are based on RNA expression and therefore are not validated at the protein level. Nevertheless, our present study offers an indication that the potential effect of SARS-CoV-2 infection on the early embryo should be further investigated using both stem cell models of the embryo and in non-human primates.

## Data accessibility

Single cell RNA-sequencing data are deposited in Gene Expression Omnibus (GEO) under accession numbers GSE136447 and GSE109555.
